# Quercetin targets VCAM1 to prevent diabetic cerebrovascular endothelial cell injury

**DOI:** 10.3389/fnagi.2022.944195

**Published:** 2022-09-01

**Authors:** Jiebin Huang, Weiwei Lin, Yuxing Sun, Qian Wang, Shidian He, Zhihua Han, Lixing Lu, Xueran Kang, Yisheng Chen, Haoran Guo, Zhiyong Cui, Chenyu Sun, Ken Go, Junyi Wu, Mengxuan Yao, Mingfeng Cao, Yuzhen Xu

**Affiliations:** ^1^Ruijin Hospital, Shanghai Jiao Tong University School of Medicine, Shanghai, China; ^2^Department of Neurosurgery, Second Affiliated Hospital of Zhejiang University School of Medicine, Zhejiang University, Hangzhou, China; ^3^Department of Otorhinolaryngology Head and Neck Surgery, Shanghai Ninth People’s Hospital, Shanghai Jiao Tong University School of Medicine, Shanghai, China; ^4^Postdoctoral Workstation, Department of Central Laboratory, The Affiliated Tai’an City Central Hospital of Qingdao University, Tai’an, China; ^5^College of Bioinformatics Science and Technology, Harbin Medical University, Harbin, China; ^6^Department of Orthopedics, Shanghai General Hospital, Shanghai Jiao Tong University School of Medicine, Shanghai Jiao Tong University, Shanghai, China; ^7^Department of Otorhinolaryngology Head and Neck Surgery, Shanghai Ninth People’s Hospital, Shanghai Jiao Tong University School of Medicine Shanghai, Shanghai, China; ^8^Department of Sports Medicine, Huashan Hospital, Fudan University, Shanghai, China; ^9^Chinese PLA Medical School, Beijing, China; ^10^Shanghai Jiao Tong University, Shanghai, China; ^11^AMITA Health Saint Joseph Hospital Chicago, Chicago, IL, United States; ^12^St. Marianna Hospital, Tokyo, Japan; ^13^Department of General Surgery, Shanghai General Hospital, Shanghai Jiao Tong University School of Medicine, Shanghai, China; ^14^Department of Orthopaedic Surgery, The Third Hospital of Hebei Medical University, Shijiazhuang, China; ^15^Department of Endocrinology, The Second Affiliated Hospital of Shandong First Medical University Tai’an, Tai’an, China; ^16^Department of Rehabilitation, The Second Affiliated Hospital of Shandong First Medical University, Tai’an, China

**Keywords:** diabetes mellitus, endothelial cell, single cell analysis, VCAM1, quercetin, brain aging, neurodegenerative diseases

## Abstract

**Introduction:**

Endothelial cells play important roles in neurodegenerative diseases caused by diabetes, therefore, we aimed at investigating the mechanisms through which endothelial cells are involved in diabetes development.

**Methods:**

Single cell analysis was performed to identify the major endothelial cell subtypes in cardiovascular tissues that are involved in diabetes development. A cell-cell communication approach was then used to identify ligand-receptor interaction pairs between these cell types. Differential expression analysis between the two experimental groups [standard chow diet group and diabetogenic diet with cholesterol (DDC) group] was used to identify diabetes-related differentially expressed genes (DEGs). The upregulated genes were used to identify candidate ligands or receptors, as well as the corresponding cell types. Cell trajectory inference was performed to identify the stage of cell development and changes in expression of candidate ligands or receptors during cell development. Gene set enrichment analysis (GSEA) was conducted to investigate the biological functions of genes of purpose. Finally, molecular dynamics simulations (MDSs) were used to predict potential drugs with the ability to target the proteins of purpose.

**Results:**

Seven cell types, including five endothelial cell subtypes (EC_1, EC_2, EC_3, EC_4, and EC_EndMT), were identified from endothelial cell-enriched single cell samples from the heart and aorta of mice. Cell-cell communication analysis revealed the potential ligand-receptor interactions between these cell types while five important ligand-receptor-associated genes, including Fn1, Vcam1, Fbn1, Col4a1, and Col4a2, were established by differential expression analysis. Among them, Vcam1 is mainly expressed in EC_EndMT and is involved in interactions between EC_EndMT and other cells. Cell trajectory extrapolation analysis revealed a shift from EC_2/EC_4 to EC_EndMT and a shift from EC_EndMT to EC_3/EC_1 during the progression of diabetes. GSEA analysis revealed that upregulation of VCAM1 may have inhibitory effects on cell growth and energy metabolism.

**Conclusion:**

EC_EndMT subtypes have a complex role in neurodegenerative diseases caused by diabetes. Through mechanisms involved in cell-cell communication, Vcam1 may play an important role in dysregulation of biological functions of EC_ EndMT. Molecular docking results of the quercetin-VCAM1 complex suggest that quercetin may be an effective drug for targeting this protein.

## Introduction

Diabetes is a metabolic disease that is associated with abnormally high blood glucose levels, which can lead to various tissue lesions, including cardiovascular, renal and neurological complications (Huo et al., 2016; Bell and Goncalves, 2020; Chao et al., 2021; Bradley et al., 2022). In recent years, the prevalence of diabetes in China has significantly increased, from 10.9 to 12.4%. When combined with the prevalence of pre-diabetes, the total prevalence can reach 50.5%, with the number of people with the disease increasing from 90 million in 2011 to 140 million in 2021, the total prevalence can reach 56% (Gong et al., 2019; Li et al., 2020; Wang et al., 2021). Among these, cognitive impairment is becoming an important complication of diabetes, what’s more, patients with diabetes are more likely to develop neurodegenerative disease as well as brain aging, such as dementia, amyotrophic lateral sclerosis, Alzheimer’s disease, and Parkinson’s disease (Biessels et al., 2006; Saltiel and Olefsky, 2017; Cheong et al., 2020). Neurodegenerative disease caused by diabetes requires long-term clinical management, therefore, it has become a major public health and social problem.

Through various mechanisms, including endothelial dysfunction, arterial damage and systemic or local inflammatory responses, diabetes has been shown to cause cerebrovascular damage, which causes neurodegenerative diseases. It can also aggravate atherosclerosis, which can in turn exacerbate ischemic diseases of the brain, such as stroke (Bradley et al., 2022; Ferket et al., 2022). Various pathways are involved in these processes, including the production of reactive oxygen species, mitochondrial dysfunction and upregulation of vasoconstrictor endothelin-1 as well as MMP-9 expressions (Bhatti et al., 2017; Hougaard et al., 2020; Urner et al., 2020; D’Onofrio et al., 2021). The current management options for diabetes include appropriate diet, exercise, medication, and insulin therapy, all of which are aimed at controlling blood glucose to achieve suitable therapeutic effects (Ling et al., 2022). Herbal medicines have received increasing attention, however, it has yet to be established whether they can improve diabetic neurodegenerative diseases. It is possible to prevent cognitive impairment and diabetes complications by using herbs (Ling et al., 2022; Tao et al., 2022). Tang G. et al. (2022) showed that naringenin alleviated p2y14 receptor-mediated diabetic cardiac autonomic neuropathy in the superior cervical ganglion to improve diabetic transitions. As an adjunct for Dasatinib, quercetin improves diabetic nephropathy (Hickson et al., 2019). Ginsenosides can reduce diabetic cerebrovascular damage by improving endothelial functions (Yang et al., 2022). Therefore, herbal medicines have positive effects with regards to management of diabetes.

In summary, diabetes can affect neurodegenerative diseases caused by diabetes through endothelial cells, and active molecules of herbs can improve diabetes by improving the function of endothelial cells. Active ingredients of Chinese medicine affect disease progression by activating downstream pathways, mainly through ligand receptor binding, to influence cellular secretory functions (Chen et al., 2022a; Zhang et al., 2022). Therefore, in order to further elaborate the possible mechanisms of the occurrence of neurodegenerative diseases and to illustrate the possible mechanisms of the involvement of Chinese medicine in the prevention of neurodegenerative diseases in diabetes, new theoretical support is provided for the precise treatment of diabetes with Chinese medicine.

## Materials and methods

### Data acquisition

Datasets were downloaded from the Gene Expression Omnibus (GEO)^1^, an international public repository for genomic data (Barrett et al., 2012). In the GEO database, the keyword “diabetes mellitus” was used to retrieve diabetes-related studies and RNA transcriptomic datasets. The data sets for bioinformatics analysis met the following criteria: i. There had a set of normal controls and ii. Their RNA transcript data could be accessed. Bulk RNA-seq data for dermal endothelial cells from four type 2 diabetic patients and six normoglycaemic controls were obtained from the GSE92724 dataset. Transcriptomic data from this dataset were stored in a TPM format and were further log-transformed for subsequent differential gene expression analyses to identify diabetes-associated differentially expressed genes (DEGs). The scRNA-seq data from the GSE169332 dataset was processed *via* the standard 10X Genomics Cell Ranger process. The GSE169332 dataset has single-cell RNA-seq data enriched with endothelial cells from the heart and aorta of Ldlr null (Ldlr^–^/^–^) mice (Zhao et al., 2021). Mice were assigned into two groups of three each. A group of mice fed on a standard chow (Chow) diet was used as the control group for no diabetic atherosclerosis; another group of mice fed on a diabetogenic diet with cholesterol (DDC) was diagnosed with diabetic atherosclerosis after 12 weeks of feeding, which was characterized by weight gain, impaired glucose tolerance and insulin sensitivity. This single-cell RNA-seq (scRNA-seq) dataset package is composed of transcripts from 1986 cells and was used to explore diabetes-related gene transcriptional differences in endothelial cells at the single-cell level.

### Single cell analysis

Cellular integration, dimensionality reduction, clustering, and cellular annotation of scRNA-seq data from this mouse were performed using standard single-cell analysis procedures based on the “Seurat” package as previous researches (Stuart et al., 2019; Chen et al., 2022b). First, quality control of scRNA-seq data was based on cell quality filtering criteria from previous studies. The exclusion criteria were: i. Cells expressing less than 300 genes or more than 4,000 genes; ii. Cells containing more than 10% of unique molecular identifiers (UMIs) derived from the mitochondrial genome; iii. Cells expressing more than 1% of hemoglobin-related genes; and iv. Gene features expressed by no more than 10 cells. A total of 1,670 cells were obtained and 12,494 genetic features were included for further analyses. Then, quality-controlled scRNA-seq data were integrated using the “SCTransform” function. Cell integration was assessed by applying the uniform manifold approximation and projection (UMAP) after running the “RunPCA” and “RunUMAP” functions (Becht et al., 2018). The “FindNeighbors” and “FindClusters” functions were used to cluster the cells and select the appropriate resolution value to establish the number of clusters.

Finally, each cell cluster was annotated using the SingleR (v. 1.4) R package and marker genes of the cell lineage (Aran et al., 2019). The first attempt involved using the SingleR package for cellular annotation of mouse scRNA-seq data based on the ImmGenData and MouseRNAseqData reference datasets, respectively. The final cell annotation was completed using previously reported cell lineage marker genes (Zhao et al., 2021). Among them, marker genes for endothelial cells are Cdh5, Pecam1, Tek, Vwf, Tie1, and Col4a1; marker genes for mesenchymal cells are Fn1, Tgfbr2, and Eln; marker genes for fibroblasts are Dcn and Col1a1 while marker genes for macrophages are Cd68, Adgre1, and Lgals3. After identification of cell types, proportions of each cell type to all cells in both groups were evaluated.

### Gene differential expression analysis

Differential expression analysis of bulk RNA-seq data from human dermal vascular endothelial cells was performed using the “limma” R package to identify diabetes-associated DEGs (Zhao et al., 2021). The up- and down-regulated mRNAs at the time of brain hemorrhage were compared to their levels 3 days after brain hemorrhage. Expressions of DEGs were considered to be significant at *p* < 0.05. Fold change (FC) > 1 was used to identify up-regulated DEGs. Endothelial cells as well as their subpopulations were extracted from the scRNA-seq data and separately analyzed for differential gene expressions at the single cell level. The “FindMarker” function from the Seurat package was used to calculate DEGs in endothelial cells between DDC and Chow groups. *p* < 0.05 and | log FC| > 0.25 were set as the thresholds for significance.

### Cell-cell communication analysis

Cell-cell communication analysis was used to explore context-dependent crosstalk across endothelial cell subtypes and other cell types during diabetes development and to identify genes that alter important physiological processes in endothelial cells (Chen et al., 2022b). CellPhoneDB, a database that can calculate ligand and receptor interactions between cells, was used to perform cell-cell communication analyses (Efremova et al., 2020). Gene expression matrices and cell type text information for each endothelial cell subtype as well as other cell types identified in the dataset were used as input files to predict potential ligand-receptor interactions between these cells. The predicted ligand-receptor interaction pairs were further screened for up-regulated genes in differential analysis of bulk RNA-seq and scRNA-seq. These up-regulated genes were included as significant genes in subsequent analyses.

### Cell trajectory inferences

Cell trajectory inference analyses of EC-enriched single-cell RNA-seq data from mouse heart and aortic tissues were performed using monocle3 (v. 1.0) and monocle2 (v. 2.4) (Trapnell et al., 2014; Qiu et al., 2017; Cao et al., 2019; Lin et al., 2021). Trajectory inference (TI), also known as pseudotime analysis, learns cell trajectories by measuring the distance of transcriptional differences between cells and further identifies different cell states. We separately performed cell trajectory inference analysis on all endothelial cells and EC_EndMT, after which we used all DEGs to learn cell trajectories. Based on test grouping and cell subtype distributions, the appropriate root was determined to calculate the pseudotime of all cells, relative to the root. Further identification of pseudotime-related genes was based on ordering of single cell pseudotimes. The DDRTree plot was used for visualization of cell trajectories as well as pseudotimes of cells.

### Gene set enrichment analysis

Gene set enrichment analysis (GSEA) is used to explore the biological processes in which important genes of interest are involved (Subramanian et al., 2005). Based on median expression values of genes of interest, samples were divided into two groups. Then, the fold change was calculated for each gene between the groups after which all genes were sorted based on FC value and used as the input file for GSEA. The “c2.cp.v7.2.symbols.gmt [Curated]” gene set in MSigDB Collections was used as the reference gene set for functional pathway annotation.^2^

### Protein and small molecule structure preparation and molecular docking

The corresponding structure information of the protein was obtained from Uniport,^3^ get the entry of P19320 and download the corresponding Alphafold2 protein structure file as previous studies (Burley et al., 2019; Tunyasuvunakool et al., 2021; Kang et al., 2022; Lu et al., 2022; Tang G.-Y. et al., 2022). Under B3LYP/6-31G* basis set conditions, the quantitative software (Orca) was used for quantum chemical optimization of the small molecule (Quercetin), involving corrections for bond lengths, bond angles, dihedral angles, and calculations of RESP2.0 fixed charges (Neese et al., 2020). The smina software was carried out to achieve small molecule docking selection (Masters et al., 2020). Briefly, the ligand with polar hydrogen addition is docked to the correctly protonated protein with a box center of (17.31, 91.29, 14.42), an x/y/z size of 126 and an exhaustiveness of 8 after which the lowest energy conformation is chosen as the final conformation to start kinetic simulation.

### Molecular dynamics simulation and energy calculation

MD simulations were performed using the Gromacs2019.4 software. Amber14sb was selected as the protein stance while the Gaff2 stance was selected for small molecules. The TIP3P water model was used to create the water box and add the sodium ion equilibrium system into the system of quercetin-VCAM1 (Van Der Spoel et al., 2005). The Coulomb force cut-off distance and van der Waals radius cut-off distance were both 1.4 nm. Finally, the system was equilibrated using a regular system (NVT) and an isothermal isobaric system (NPT), followed by a 100 ns MD simulation at room temperature and pressure. During MD simulations, the involved hydrogen bonds were constrained using the LINCS algorithm with an integration step of 2 fs. Electrostatic interactions were calculated using the Particle-mesh Ewald (PME) method with a cut-off value of 1.2 nm. The non-bond interaction cut-off value was 10 Å. The V-rescale temperature coupling method was used to control the simulated temperature to 300 K while the Berendsen method was used to control the pressure to 1 bar. Finally, a 100 ns simulation of the finished MD of the protein-ligand complex system was performed. Visualization of simulation results was performed using the Gromacs embedded program and Pymol 2.4. Free binding energy between protein and ligands was calculated using the MM/GBSA method (Valdés-Tresanco et al., 2021). In this study, the 25–30 ns MD trajectory was used in calculation, as follows:


$$\Delta \,G_{\text{bind}}=\Delta \,G_{\text{complex}}-\left(\Delta \,G_{\text{receptor}}+\Delta \,G_{\text{ligand}}\right)$$

$$=\Delta \,E_{\text{internal}}+\Delta \,E_{\text{VDW}}+\Delta \,E_{\text{elec}}+\Delta \,G_{\text{GB}}+\Delta \,G_{\text{SA}}$$

Whereby, ΔE_internal_ represents internal energy, ΔE_VDW_ represents van der Waals interactions and ΔE_elec_ represents electrostatic interactions. Internal energies include Ebond, Eangle, and Etorsion; ΔG_GB_ and ΔG_GA_ are collectively referred to as free energy of solvation. Among them, G_GB_ is the polar solvation free energy while G_SA_ is the non-polar solvation free energy. For ΔG_GB_, the GB model developed by Nguyen et al. (2015) was used for calculations (igb = 8). The non-polar solvation free energy (G_SA_) was calculated as the product of surface tension (γ) and solvent accessible surface area (SA), G_SA_ = 0.0072 × ΔSASA (Weiser et al., 1999). We ignored entropy variation in this study because of its high consumption of computational resources and low accuracy.

### Statistical analysis

All plotting was done using the R software (v. 4.0.2). The “VennDiagram” R package was used for plotting the Venn diagrams. Differences in proportions of cell types between the two groups were evaluated by the chi-square test. *p* ≤ 0.05 was the threshold for statistical significance, unless otherwise stated.

## Results

### The endothelial cell subtypes

**FIGURE 1:**
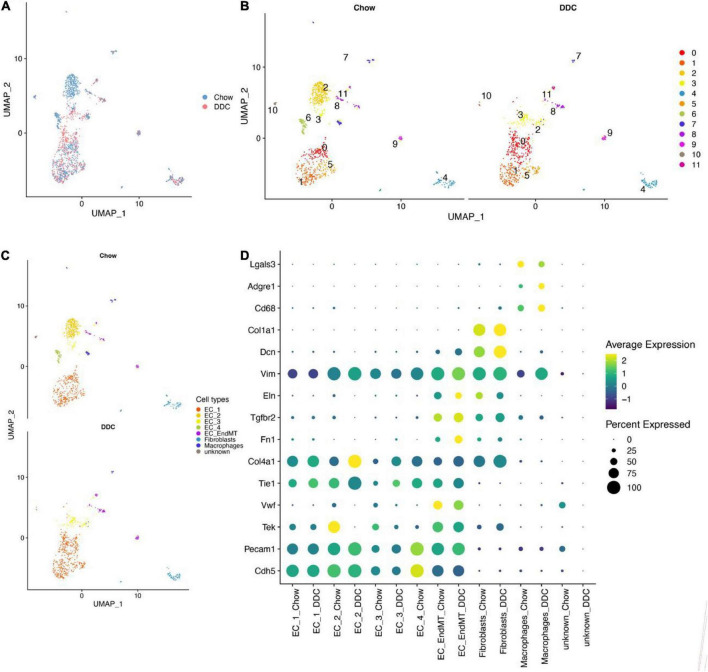
EC-enriched single-cell RNA-seq analysis of mouse heart and aorta. **(A)** UMAP plot showed cell aggregation in the Chow group and DDC group. The colors represent different groups. **(B)** UMAP plot showed the clusters of cells from the Chow and DDC groups after cell clustering. Different colors indicated different cell clusters; the Chow group showed 12 clusters of cells, while the DDC group showed 11 clusters. **(C)** UMAP plot showed 7 cell types identified in the Chow and DDC groups, among others, containing 5 endothelial cell subtypes (EC_1, EC_2, EC_3, EC_4, EC_EndMT). **(D)** Dot plots showed the expressions of selected marker genes for each cell type between the groups. Dot sizes indicated the percentage of cells expressing each gene while dot color represented the average expression level in each group. The vertical axis showed the endothelial cell marker genes (Cdh5, Pecam1, Tek, Vwf, Tie1, and Col4a1), mesenchymal marker genes (Fn1, Tgfbr2, and Eln), fibroblast marker genes (Dcn and Col1a1) and macrophage marker genes (Cd68, Adgre1, and Lgals3).

The distributions of characteristics in EC-enriched single cells from mouse heart and aorta before and after quality control are shown in Supplementary Figure 1. Single cell transcript data integration (Figure 1A and Supplementary Figure 2A) and cell clustering were performed and 12 cell clusters were identified (Supplementary Figure 2B), with 12 clusters in the Chow group and 11 clusters in the DDC group (Figure 1B). Automatic annotation of cell clusters using SingleR revealed three main cell types; endothelial cells, fibroblasts, and monocytes/macrophages (Supplementary Figure 2C). Then, expressions of marker genes for appeal cell lineages were analyzed in each cell cluster (Supplementary Figures 2D, 3). Cluster 4 was identified as fibroblasts, due to its high expressions of fibroblast marker genes (Dcn and Col1a1). Cluster 7 was identified as macrophages, due to its high expression of macrophage marker genes (Cd68, Adgre1, and Lgals3). Apart from cluster 10, the other clusters were identified as endothelial cells due to their high expressions of endothelial cell marker genes (Cdh5, Pecam1, Tek, Vwf, Tie1, and Col4a1). Clusters 8, 9, and 11 were identified as endothelial-mesenchymal transition (EndMT) ECs, based on their expressions of mesenchymal marker genes (Fn1, Tgfbr2, and Eln). Ultimately, 7 cell types, including five EC subtypes (EC_1, EC_2, EC_3, EC_4, and EC_EndMT) were identified from this single cell RNA-seq data (Figure 1C). Expressions of selected marker genes for each cell type were consistent between the groups (Figure 1D).

### Proportions of each endothelial cell subtype differed between the two groups

**FIGURE 2:**
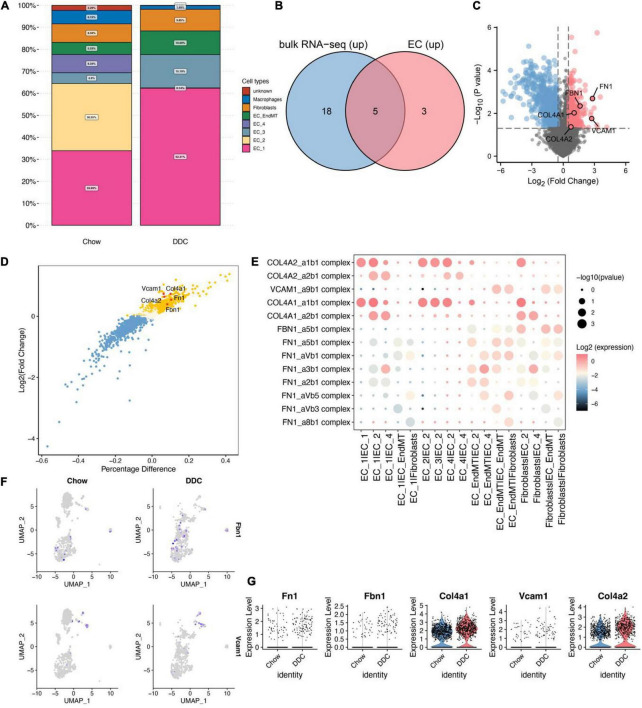
Identification of up-regulated genes in the DM group based on cellular communication ligand-receptor analysis. **(A)** Proportions of each cell type in Chow and DDC groups. **(B)** Differential analysis based on bulk RNA-seq data and endothelial cell scRNA-seq data revealed that 23/8 genes from predicted ligand-receptor pairs were upregulated in the DM group. Intersection analysis revealed five up-regulated important genes in the DM group. **(C)** Volcano plot showed the results of differential analysis of the five important genes (*FN1*, *VCAM1*, *FBN1*, *COL4A1*, and *COL4A2*) based on bulk RNA-seq data. **(D)** The results showed differential analysis of the 5 important genes based on scRNA-seq data from endothelial cells. **(E)** Dot plots showed that ligand-receptor pairs contain the five important genes in cell receptor-ligand interaction analysis and corresponding pairs of interacting cell types. **(F)** UMAP plots showed the expressions of Fbn1 and Vcam1 in endothelial cells. **(G)** Violin plots showed the expressions of the 5 important genes between the Chow and DDC groups.

The proportion of EC_1 was highest in both the Chow (33.89%) and DDC (62.31) groups and was significantly higher in DDC group, relative to Chow group (Figure 2A, *p* < 0.001). Moreover, EC_3 levels were markedly high in the DDC group than in the Chow group (15.15% vs. 4.9%, *p* < 0.001). However, EC_2 and EC_4 were essentially only present in the Chow group and, in particular, EC_2 was only present in the Chow group. The abundance of EC_EndMT was significantly higher in the DDC group, relative to the Chow group (10.69% vs. 5.53%, *p* < 0.001). Differences in proportions of fibroblasts between the groups were insignificant (*p* = 0.38). These findings show that the abundance of each endothelial cell subtype differed between the DDC and Chow groups, suggesting altered endothelium functions during diabetes progression.

### Ligand-receptor interactions in endothelial cells

Differential analysis of bulk RNA-seq data based on dermal endothelial cells revealed 981 upregulated genes in DM, of which 23 were included in the predicted ligand-receptor pairs from cell-cell communication analysis. Based on differential analysis of endothelial cell scRNA-seq data and cell-cell communication analysis, eight up-regulated genes were included in the predicted ligand-receptor pairs. Intersection analyses of these two sets of genes revealed five endothelium-associated upregulated genes important in DM development (Figure 2B). Differential expressions of the five important genes (Fn1, Vcam1, Fbn1, Col4a1, and Col4a2) in dermal endothelial cells bulk RNA-seq and endothelial cell scRNA-seq are presented in volcano and scatter plots, respectively (Figures 2C,D). During DM development, these five genes are involved in dysregulation of endothelial cell functions through ligand-receptor interactions. Ligand-receptor pairs in which these five genes are located, and the cell type pairs in which they correspond to, are shown in Figure 2E. Among them, Fbn1 and Vcam1 were enriched in interactions of EC_EndMT and fibroblasts. Vcam1 was highly expressed in EC_EndMT (Figures 2F,G). In summary, the elevated levels and dysregulated functions of EC_EndMT may play important roles in diabetes development, while Vcam1 may be involved in mediating the functions of EC_EndMT through ligand-receptor interactions.

### Expressions of the 5 genes in various endothelial cell subtypes

The abundance of EC_1 and EC_3 subtypes were high in the DDC group, relative to the Chow group. Differential expression analysis revealed that Fbn1, Col4a1 and Col4a2 expressed by EC_1 were upregulated in DDC group, compared to Chow group (Supplementary Figures 4A,B); Expressions of Fn1, Col4a1 and Col4a2 were upregulated in EC_3 (Supplementary Figures 4C,D). The EC_2 and EC_4 isoforms are predominantly present in the DDC group and are characterized by expressions of Col4a1 and Col4a2 (Supplementary Figures 4E,F). Therefore, the EC_2 and EC_4 endothelial cell subtypes may play important roles in maintaining normal endothelial tissue functions while EC_1 and EC_3 may be involved in diabetes progression. In addition, ligand-receptor interaction pairs related to Fbn1 and Fn1 may play a role in conversion of the biological functions of endothelial cells.

### Conversion of each endothelial cell subtype between Chow and diabetogenic diet with cholesterol groups

**FIGURE 3:**
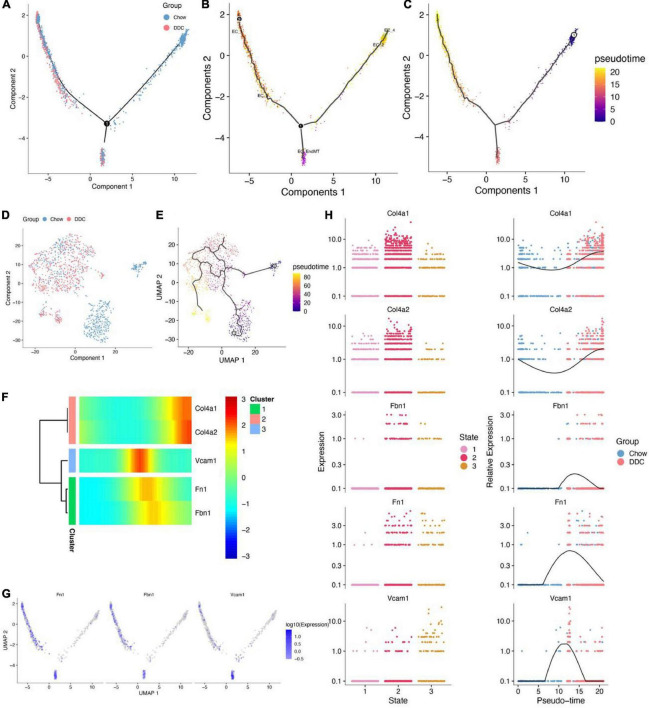
Cell trajectory analysis of five endothelial cell subtypes. **(A)** DDRTree plot of endothelial cells between Chow and DDC groups. **(B)** DDRTree plot showed the various cell subtypes of endothelial cells. **(C)** DDRTree plot showed the pseudotime of endothelial cells. **(D)** UMAP plot showed endothelial cells between Chow and DDC groups based on Monocle 2. **(E)** UMAP plot of pseudotime of endothelial cells. **(F)** Five important genes (Col4a1, Col4a2, Vcam1, Fn1, and Fbn1) were associated with pseudotime and cell trajectory differentiation of endothelial cells. **(G)** DDRTree plots of expressions of Fn1, Fbn1, and Vcam1 in endothelial cells. **(H)** Five important genes were differentially expressed between cell states and their expressions were accompanied by pseudotime. Expressions of Fbn1 and Vcam1 were the highest in the middle segment of the cell trajectory based on pseudotime.

All endothelial trajectories between the groups were inferred (Trajectory inference) based on different transcriptional characteristics of endothelial cells and the group, endothelial cell subtypes and pseudo-temporal characteristics were projected onto the DDRTree plot (Figures 3A,C). The UMAP was used for further visualization of the distribution of each endothelial cell subtype (Supplementary Figure 5A). The DDRTree plot showed the 3 stages (states) and pseudotimes (Supplementary Figures 5B–D) of the endothelium. The cell state in which EC_2 and EC_4 were unique to the Chow group is defined as the root. Thus, it could be postulated that endothelial cells transition from EC_2/EC_4 to EC_EndMT and subsequently to EC_3/EC_1 (Figure 3B). The UMAP showed the grouping of endothelial cells (Figure 3D) and the transformation trajectory of endothelial cells (Figure 3E). The appearance of the EC_EndMT isoform throughout the endothelial cell trajectory could be seen in multiple locations and cellular stages, indicating its complex role in progression of diabetes. Changes in expressions of the top 50 genes that were associated with pseudo-temporal sorting of cells were shown in Supplementary Figure 5E. Among them, Vcam1, Fn1, and Fbn1 were found to be upregulated in the pseudo-time-based intermediate segment of the trajectory (Figures 3F–H), while Vcam1 was predominantly expressed in the EC_EndMT isoform.

### Trajectory inference for the endothelial-mesenchymal transition endothelial cell subtype

**FIGURE 4:**
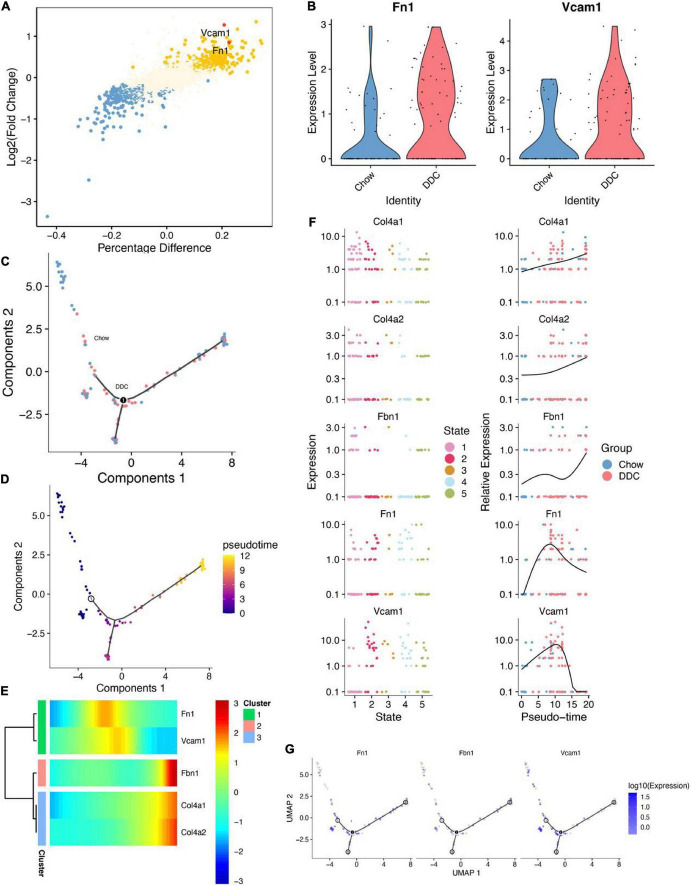
Cell trajectory inference analysis of EC_EndMT. **(A)** The results showed differential analysis of Vcam1 and Fn1. **(B)** Vcam1 and Fn1 were upregulated in DDC group compared to Chow group. **(C)** DDRTree plot showed the distributions of EC_EndMT subtypes between Chow and DDC groups. **(D)** DDRTree plot of pseudotime of EC_EndMT subtypes. **(E)** Expressions of 5 important genes in EC_EndMT were accompanied by changes in the sorting of pseudotime. **(F)** Expressions of these five important genes between cell states (left), and their expressions were accompanied by changes in the sorting of pseudotime (right). **(G)** DDRTree plots for Fn1, Fbn1, and Vcam1 expressions in EC_EndMT.

Cellular trajectory analysis was performed to investigate the role of the EC_EndMT subtype in diabetes development. First, differential expression analysis showed that Vcam1 and Fn1 expressed by EC_EndMT were upregulated in the DDC group (Figures 4A,B). Then, EndMT ECs were subjected to trajectory learning and cell stage identification (Supplementary Figure 5F). DDRTree plots showed the distributions of trajectories of EC_EndMT, along with the groups in which they belong to and their pseudotime (Figures 4C,D). Vcam1 and Fn1 were highly expressed at intermediate pseudo-time periods (Figures 4E,F). Pseudo-time periods corresponded to cellular stages at states 2, 3, and 4 (Figure 4G). These findings imply that Vcam1 is involved in dysregulation of biological functions of EC_EndMT through cell-cell communication during diabetes development.

### VCAM1 has inhibitory roles on cell growth and energy metabolism

**FIGURE 5:**
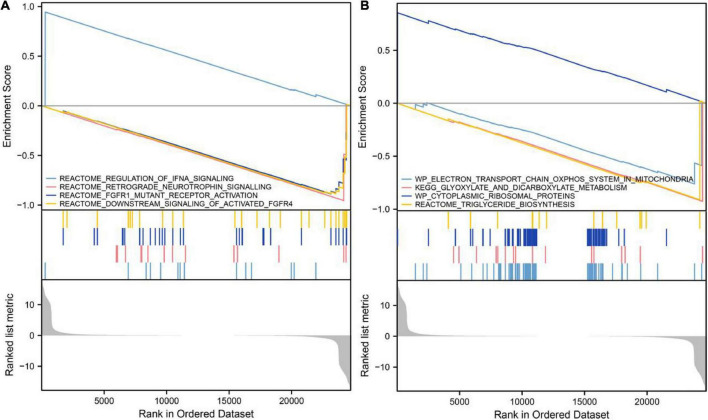
Gene set enrichment analysis of VCAM1. **(A)** Upregulation of VCAM1 was accompanied by inhibition of pathways associated with promotion of cell growth. **(B)** Upregulation of VCAM1 was accompanied by inhibition of energy and metabolism-related pathways.

GSEA showed that in samples with upregulated VCAM1, IFNA signaling was upregulated, while retrograde neurotrophin signaling, FGFR1 mutant receptor activation and downstream signaling of activated FGFR4 pathways associated with cell growth were downregulated (Figure 5A). In addition, cytoplasmic ribosomal proteins were upregulated, while energy and metabolism-related pathways, such as electron transport chain: OXPHOS system in the mitochondria and dicarboxylate metabolism, and triglyceride biosynthesis were downregulated (Figure 5B). These findings imply that elevated VCAM1 inhibits cell growth and energy metabolism, thereby, playing a role in diabetes development.

### Analysis of molecular dynamics simulation results

**FIGURE 6:**
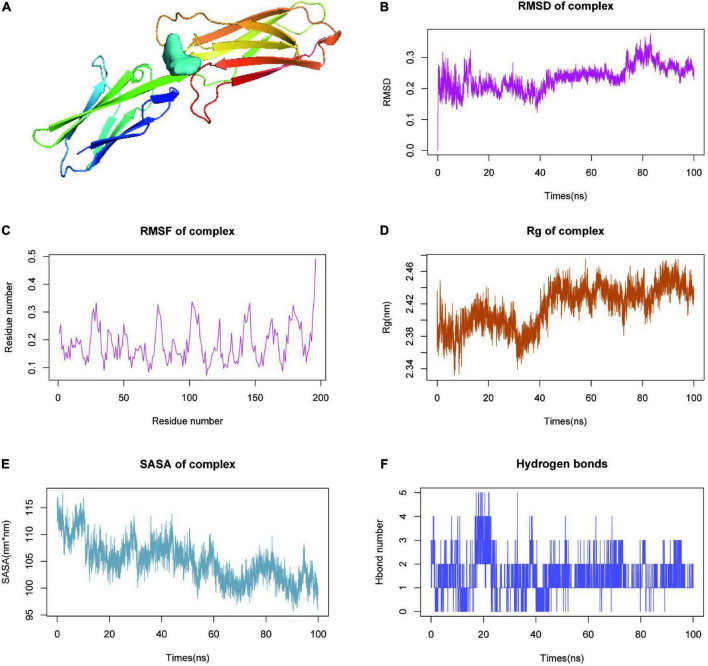
Molecular dynamics simulation (MDS) analysis of VCAM1-quercetin. **(A)** Molecular docking results of quercetin-VCAM1 complex. **(B)** Root-mean-square deviation of quercetin-VCAM1 complex MDS. **(C)** Root-mean-square fluctuation plot of quercetin-VCAM1 complex MDS. **(D)** Plot of Rg changes in MDS of quercetin-VCAM1 complex. **(E)** SASA changes of proteins in quercetin-VCAM1 complex MDS 0–100 ns. **(F)** Changes in hydrogen bonding in steady state of quercetin-VCAM1 complex.

Findings from molecular docking of the quercetin-VCAM1 complex were shown in Figure 6A. Molecular dynamics simulation (MDS) is an important method for studying the stability and kinetic characteristics of complexes in aqueous solutions. Atomic root mean square deviation (RMSD) provides a measure of the stability of the system. RMSD values fluctuated at roughly 5, 50, and 82 ns, where they started as a result of transient instability obtained from balanced docking, and then entered a steady state at 20 ns and a final steady state at 80 ns (Figure 6B). Root mean square fluctuations (RMSF) allow the system to be observed as local sites change configuration during simulation (Figure 6C). With a cut-off value of 0.25 fluctuations, amino acids 26–32, 75–78, 101–107, 142–146, and 176–185 could be seen to be highly volatile. The radius of gyration (Rg) is an important indicator for evaluating the tightness of the structure of the system. Rg analysis of the Quercetin-VCAM1 complex revealed a fluctuation at 5/50 ns each, corresponding to the ripple in RMSD, indicating that the system is undergoing a transition from multiple instabilities to platforms at this moment (Figure 6D). A steady decrease in Solvent Accessible Surface on Protein Surface (SASA) was found for proteins from 0 to 100 ns, indicating favorable binding and progressive protein tightening (Figure 6E). The hydrogen bonding change curve showed that the quercetin-VCAM1 complex has 2–3 hydrogen bonds in the steady state (Figure 6F). The above analyses imply that the quercetin-VCAM1 complex system becomes more stable after a series of transformations as MDS proceeds. The middle and both ends of the ligand-bound protein have red areas, therefore, this kinetic simulation causes a distal change in protein conformation.

### Molecular dynamics simulation of secondary structures and binding free energy analysis

**FIGURE 7:**
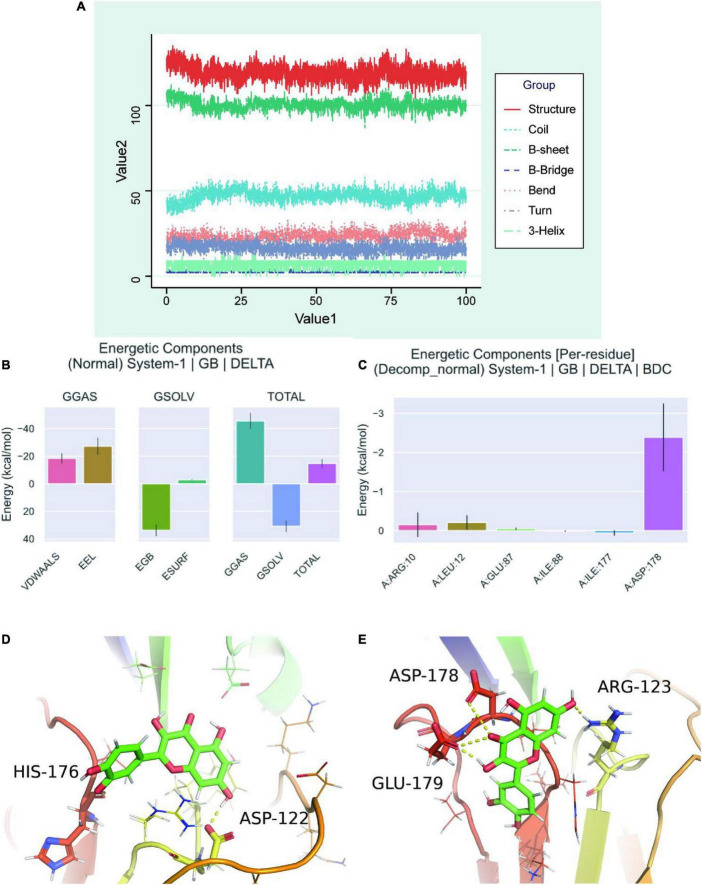
Molecular dynamics simulation analysis of secondary structure. **(A)** A graph showed the change of individual secondary structures of proteins over time in MDS system. **(B)** Free energy of binding between protein-ligand complexes. VDWAALS, “van der Waals energy”; Eel, “Electrostatic energy”; EGB, “Polar solvation energy”; ESURF, “Non-polar solvation energy”; GGAS, “Total gas phase free energy”; GSOLV, “Total solvation free energy”; TOTAL, The free energy item: “GSOLV + GGAS.” **(C)** Relationship between each contact residue with binding energy. **(D)** Binding of quercetin-VCAM1 complex at critical frame of 22 ns. **(E)** Binding of quercetin-VCAM1 complex at critical frame of 73.4 ns.

Secondary structure analysis based on MDS revealed that this simulation involves structures such as coil, B-sheet, B-bridge, bend, turn, and 3-helix, where the overall number of structures becomes less as simulation progresses, b-sheet and 3-helix decreases, but the number of coil and bend increases (Figure 7A). After calculating the free energy of binding between protein-ligand complexes, we calculated the differences in free energy of binding between two solvated molecules in the bound and unbound states and compared the free energies of different solvated conformations of the same molecule (Figure 7B). Analysis of the data on variations of binding free energy with MDS revealed that the energies were all negative in TOTAL free energy, suggesting a strong possibility of interactions between the protein and the small molecule. To break down each small item, “GGAS” represents the gas-phase free energy, which is negative, and is calculated by combining VDWAALS (“van der Waals energy”) and EEl (“Electrostatic energy”). VDWAALS and Eel are both < 0, indicating that both hydrophobic and electrostatic interactions contribute to binding, while electrostatic interactions inhibit binding. In contrast, GSOLV represents “Total solvation free energy” which is positive, indicating unfavorable binding. GSOLV resulted from interactions of ESURF (“Non-polar solvation energy”) and EGB (“Polar solvation energy”), with positive EGB values indicating that polar solvation is not conducive for binding. Breaking down each contact residue, it was shown that ILE-177 acts as a barrier to binding while ARG-10, LEU-12, GLU-87, and ASP-178 promote binding (Figure 7C). The contribution of ASP-178 was significant, thus, further keyframe analysis was performed and 22 ns as well as 73.4 ns were selected as key frames for analysis. At 22 ns, HIS-176, and ASP-122 were linked by hydrogen bonding polarity from the head and tail of the small molecule, respectively. Both are small molecules with H as the hydrogen donor and the protein as the hydrogen acceptor (Figure 7D). At 73.4 ns, after a 50 ns perturbation, the small molecule finally proceeded to the steady state (Figure 7E). At this point, the complex system remained in the previous cavity pocket while the small molecule changes position, with ARG-123 inheriting the previous active role of ASP-122 having hydrogen bonding with the small molecule. The GLU-179 and ASP-178 interacted with the small molecule to jointly stabilize it, where GLU-179 and Arg-123 act as hydrogen co-donors and ASP-178 acts as a hydrogen acceptor.

In summary, VCAM1 plays an important role in diabetic neurovascular complications through the endothelial cell subtype EC_EndMT, and quercetin can prevent diabetic neurovascular complications by targeting VCAM1.

## Discussion

We identified the endothelial cell types associated with neurodegenerative diseases of diabetes and further identified five important genes, *FN1*, *VCAM1*, *FBN1*, *COL4A1*, and *COL4A2*. The *FN1* gene encodes the fibronectin protein, which is involved in cell adhesion and migration. FN1 is involved in the development and progression of various diseases, including spondyloepiphyseal dysplasia, corner fracture type and glomerulopathy with fibrin deposition (Zollinger and Smith, 2017). Moreover, it plays a roles in progression of diabetic nephropathy, however, the involved mechanisms are unclear (Yang et al., 2021). In addition, FN1 can influence the development of amyotrophic lateral sclerosis, one of the neurodegenerative diseases, by affecting the NF-κB pathway (Lin et al., 2020). *FBN1* encodes fibrillin, which can act as a structural component of calcium-bound microfibrils that provide load-bearing structural support in elastic and inelastic connective tissues. The FBN1-associated diseases include Marfan syndrome and skin stiffness syndrome (Judge and Dietz, 2005; Sakai et al., 2016; Yu et al., 2020). It has been reported that FBN1 can initiate the development of obesity-induced diabetes (Hoffmann et al., 2020). *COL4A1* and *COL4A2* can encode different subunits of type IV collagen, a major structural component of the mucosa, and are associated with various diseases, including retinal artery tortuosity, cerebral small vessel disease with or without ocular abnormalities, cerebral small vessel disease and cerebral hemorrhage, which is closely associated with vascular disease (Volonghi et al., 2010; Kuo et al., 2012; Meuwissen et al., 2015). COL4A1 is involved in diabetic nephropathy while COL4A2 is involved in diabetic cardiovascular disease development (Adams et al., 2014; Rizvi et al., 2014). VCAM1, a member of the Ig superfamily, encodes a cell surface salivary gland glycoprotein expressed by endothelial cells, whose main function is to participate in cell adhesion and signaling, and possibly in neurodegenerative diseases of diabetes (Willeit et al., 2017; Kong et al., 2018; Niedzielski et al., 2020). We found that Fbn1 interact with Vcam1 mainly at EC_EndMT and fibroblasts. Further cell trajectory studies revealed that Vcam1 may play important roles in dysregulation of biological functions of EC_EndMT *via* cell-cell communication. Other studies have suggested that Vcam1 could be a marker for transitional obesity and diabetic nephropathy (Fadel et al., 2021). In the presence of hyperinsulinemia in type 2 diabetes, it activates the MAPK signaling pathway, which in turn activates the expressions VCAM1 and E-selectin and induces the production of ROS, involved in vascular damage in diabetes, including the neurodegenerative diseases (Potenza et al., 2009). We found that VCAM1 can inhibit endothelial cell growth and energy metabolism, suggesting that VCAM1 can promote the development of neurodegenerative diseases of diabetes. Therefore, we used VCAM1 as a site for action and performed MDSs of its potential action as a drug and realized that quercetin has a large binding potential to VCAM1.

Quercetin, a major constituent of many traditional Chinese medicines, has certain anti-tumor and anti-platelet aggregation effects. It is a flavonol compound with multiple biological activities, which can exert antioxidant effects by activating the NrF-2-ARE pathway or by promoting the expressions of antioxidant proteins (CAT and SOD) to improve the symptoms of several diseases, including neurodegenerative diseases, tumors, inflammatory diseases, obesity and diabetes (Wang et al., 2013; Kumar et al., 2016; Mukhopadhyay et al., 2018; Yarahmadi et al., 2018; Ebrahimpour et al., 2020; Abdou et al., 2022). Quercetin suppresses inflammation by reducing the expressions of pro-inflammatory factors, including IL-6 and IL-1β (Cheng et al., 2019). In addition, it can reduce MDA and NO levels, inhibit PI3K/PKB expressions to regulate glucose metabolism and reduce oxidative damage, as well as inhibit the NF-κB signaling pathway to improve islet functions. Above all, quercetin could improve the neurovascular symptoms of diabetes and reduce the incidence of neurodegenerative diseases in diabetic patients. Therefore, quercetin is a potential treatment option for type 2 diabetes and the complications of diabetes (Mukhopadhyay et al., 2018; Pathak et al., 2018). In addition, quercetin suppressed VCAM1 as well as ROS levels and delayed the progression of neurovascular complication of diabetes (Lotito et al., 2011; Wei et al., 2020). These studies suggest that quercetin has some complication-reducing effects in diabetes, and that for neurodegenerative diseases, quercetin can improve diabetic neurovascular damage by targeting VCAM1.

In summary, we identified key cells and factors involved in the progression of diabetes and its complications through single cell sequencing analysis data for the first time, providing a new theoretical basis for the diagnosis and treatment of diabetes. However, this study has some limitations. *In vivo* and *ex vivo* assays should be performed to further investigate the molecular mechanisms of action of quercetin.

## Conclusion

We identified a complex role for EC_EndMT subtypes in progression of diabetes. Furthermore, it revealed the potential role in the development of neurodegenerative diseases of diabetes and developed a new Chinese medicine against the target, provided theoretical support for traditional Chinese medicine treatment of neurovascular complications. Through mechanisms involved in single cell analysis and cell-cell communication, VCAM1 may play an important role in neurodegenerative diseases of diabetes, and quercetin may be an effective drug for precise treatment of neurodegenerative diseases.

## Data availability statement

The original contributions presented in this study are included in the article/Supplementary material, further inquiries can be directed to the corresponding author/s.

## Author contributions

JH, WL, YS, QW, SH, ZH, LL, and XK: conceptualization, methodology, software, investigation, formal analysis, and writing—original draft. YC, HG, and ZC: data curation and writing—original draft. CS, KG, JW, MY, and MC: visualization and investigation. YX: conceptualization, funding acquisition, resources, supervision, and writing—review and editing. All authors contributed to the article and approved the submitted version.

## Footnotes

1
https://www.ncbi.nlm.nih.gov/geo/

2
https://www.gsea-msigdb.org/gsea/msigdb/

3
https://www.uniprot.org/uniprot

## Supplementary material

Supplementary Figure 1 Quality control of single-cell RNA-seq data. **(A)** Maps showed the distribution of the main features of cells before quality control. Red dashed line represented the threshold used to perform data filtering. **(B–E)** Data quality assessment after single-cell RNA-seq data filtering.

Supplementary Figure 2 Cell clustering and annotation process for single cell analysis. **(A)** UMAP plot showed cell aggregation in Chow and DDC groups. **(B)** UMAP plot showed the number and distribution of cell clusters after cell clustering. **(C)** UMAP plots showed the initial cell annotation of cell clusters by application of SingleR. The left side was based on MouseRNAseqData reference dataset, while the right side was based on ImmGenData reference dataset. **(D)** Violin plots of cell cluster and lineage marker gene.

Supplementary Figure 3 Visualization display of expressions of endothelial cell, mesenchymal, fibroblast and macrophage marker genes. Cdh5, Pecam1, Tek, Vwf, Tie1, and Col4a1 were marker genes for endothelial cell; Fn1, Tgfbr2, and Eln were marker genes for mesenchymal: Dcn and Col1a1 were marker genes for fibroblast; Cd68, Adgre1, and Lgals3 were marker genes for macrophage.

Supplementary Figure 4 Differential expression analysis of the five important genes in endothelial cell subtypes (EC_1, EC_2, EC_3, and EC_4). **(A,B)** Differential expression analysis of genes in EC_1. **(C,D)** Differential expression analysis of genes in EC_3. **(E)** Expressions of the five genes in EC_2. **(F)** Expressions of the five genes in EC_4 in the Chow group.

Supplementary Figure 5 Single-cell trajectories inferential analysis of endothelial cells. **(A)** UMAP plot showed individual cell types in cell clustering based on Monocle 2. **(B)** DDRtree plots showed the 3 cell states in endothelial cells. **(C)** DDRTree plot showed pseudotime values for endothelial cells. **(D)** The cell subtypes of endothelial cells in DDRtree plot. **(E)** Changes in expressions of the top 50 genes that were most associated with sorting of pseudotime. **(F)** DDRtree plot showed 5 cell states in the EC_EndMT.

## Abbreviations

GEO: Gene Expression Omnibus
DEGs: Differentially expressed genes
GSEA: Gene Set Enrichment Analysis
scRNA-seq: Single cell RNA sequencing
EndMT: Endothelial-mesenchymal transition
EC: Endothelial cell.
