# Supplementary figures and images for: Quercetin targets VCAM1 to prevent diabetic cerebrovascular endothelial cell injury
Source: Front Aging Neurosci. 2022 Sep 1;14:944195. doi: 10.3389/fnagi.2022.944195 (PMC9475220; doi:10.3389/fnagi.2022.944195)

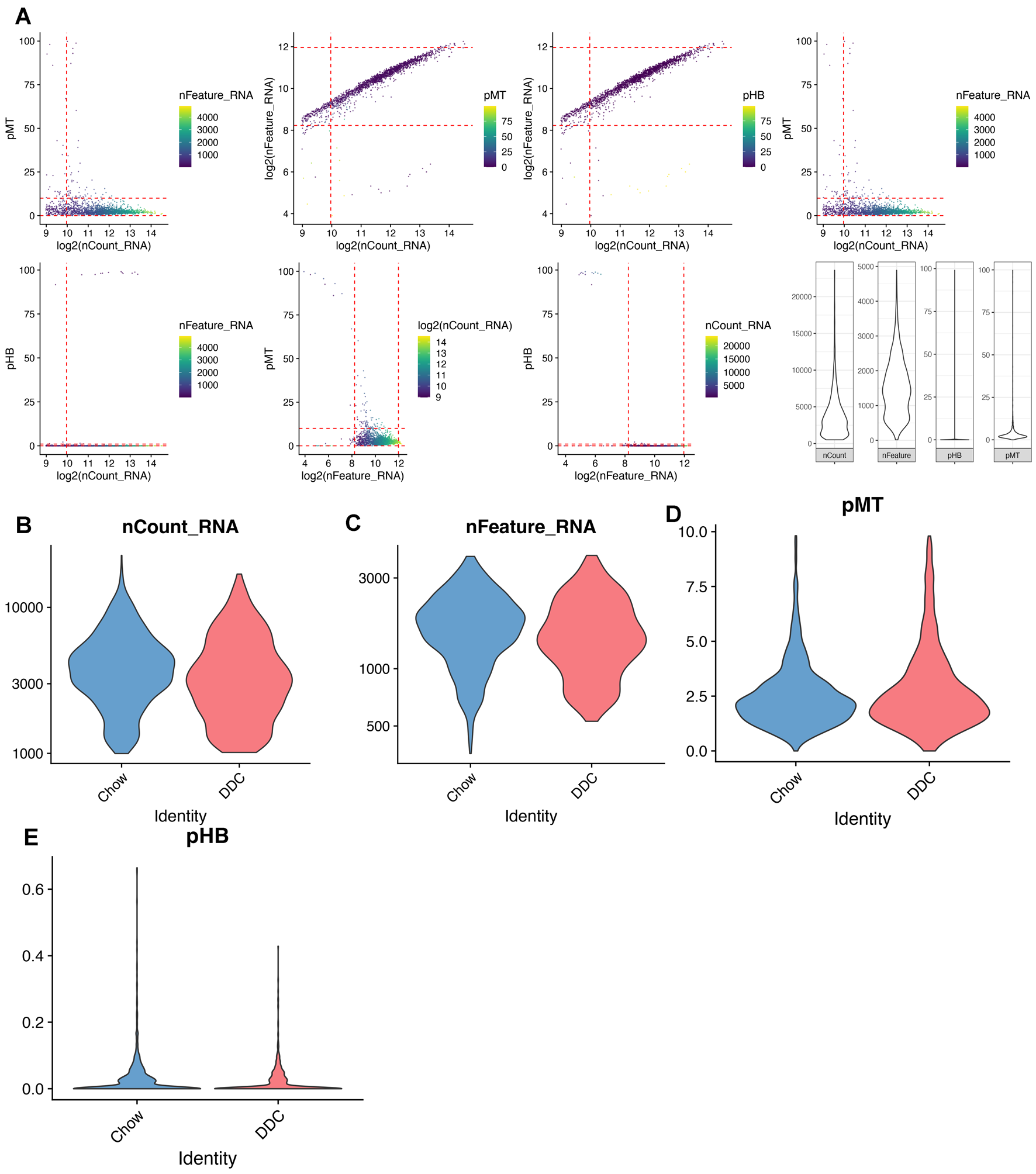

Supplement: Supplementary Figure 1 — Quality control of single-cell RNA-seq data. (A) Maps showed the distribution of the main features of cells before quality control. Red dashed line represented the threshold used to perform data filtering. (B–E) Data quality assessment after single-cell RNA-seq data filtering. [file Image_1.TIF]

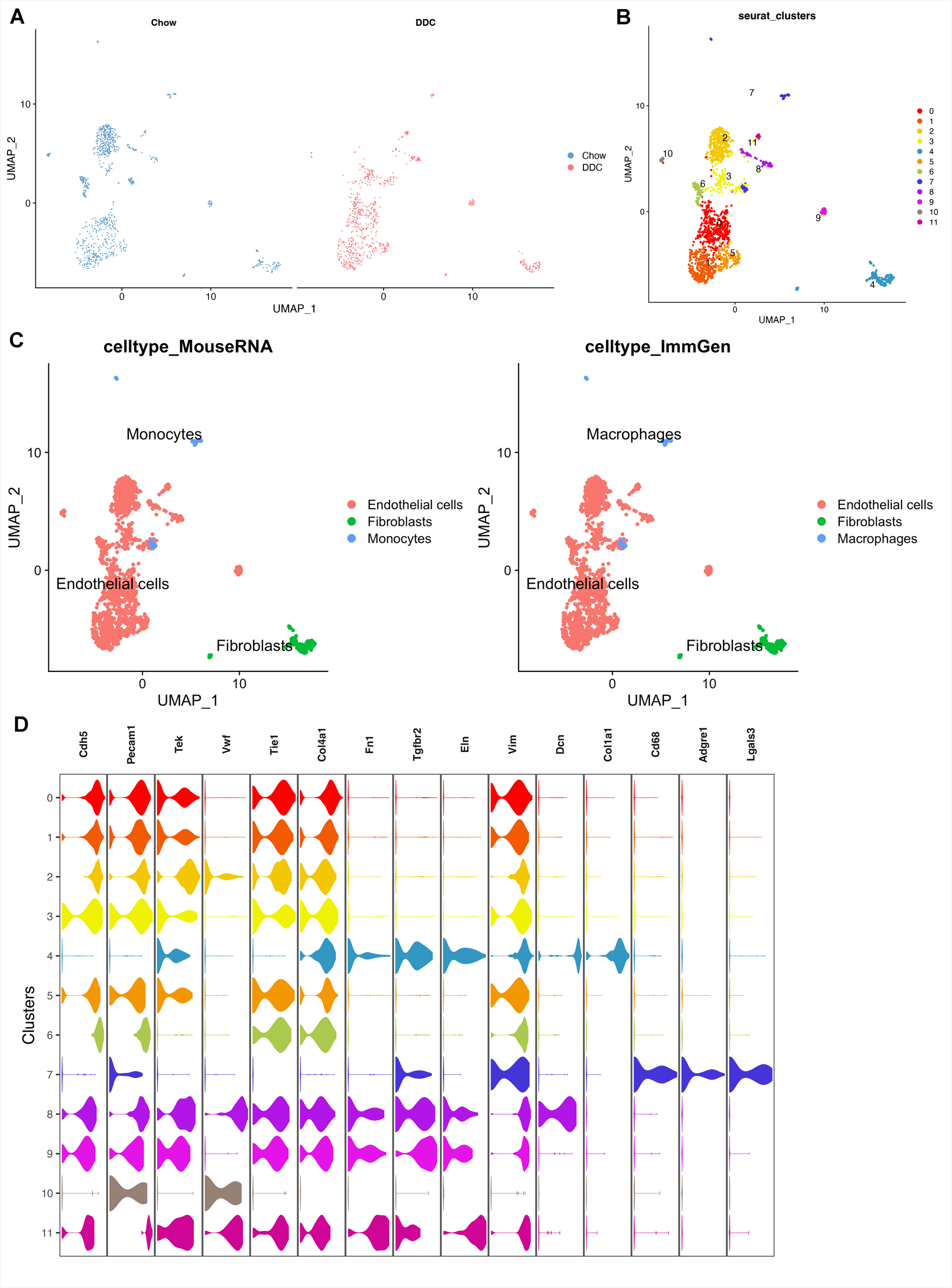

Supplement: Supplementary Figure 2 — Cell clustering and annotation process for single cell analysis. (A) UMAP plot showed cell aggregation in Chow and DDC groups. (B) UMAP plot showed the number and distribution of cell clusters after cell clustering. (C) UMAP plots showed the initial cell annotation of cell clusters by application of SingleR. The left side was based on MouseRNAseqData reference dataset, while the right side was based on ImmGenData reference dataset. (D) Violin plots of cell cluster and lineage marker gene. [file Image_2.TIF]

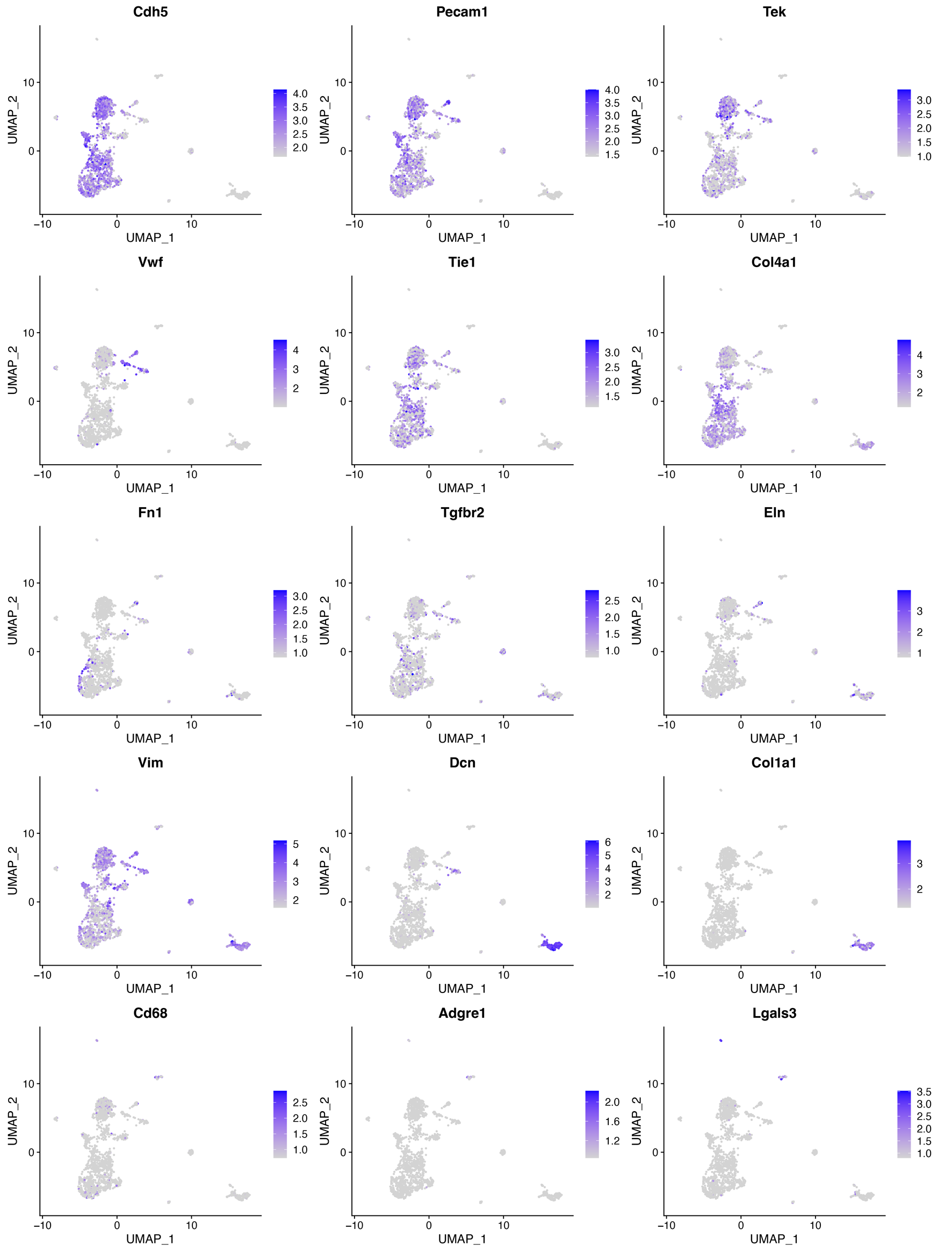

Supplement: Supplementary Figure 3 — Visualization display of expressions of endothelial cell, mesenchymal, fibroblast and macrophage marker genes. Cdh5, Pecam1, Tek, Vwf, Tie1, and Col4a1 were marker genes for endothelial cell; Fn1, Tgfbr2, and Eln were marker genes for mesenchymal: Dcn and Col1a1 were marker genes for fibroblast; Cd68, Adgre1, and Lgals3 were marker genes for macrophage. [file Image_3.TIF]

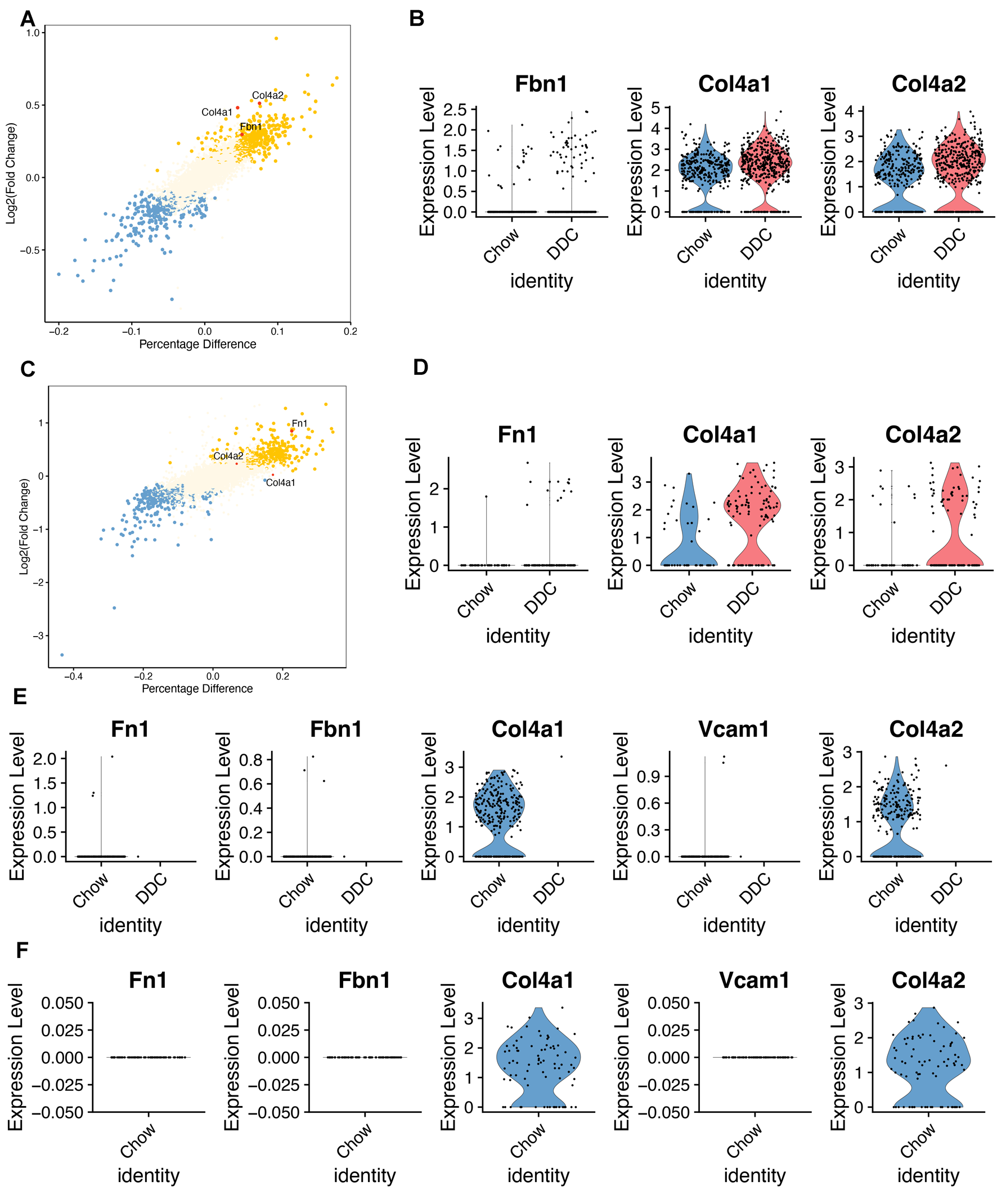

Supplement: Supplementary Figure 4 — Differential expression analysis of the five important genes in endothelial cell subtypes (EC_1, EC_2, EC_3, and EC_4). (A,B) Differential expression analysis of genes in EC_1. (C,D) Differential expression analysis of genes in EC_3. (E) Expressions of the five genes in EC_2. (F) Expressions of the five genes in EC_4 in the Chow group. [file Image_4.TIF]

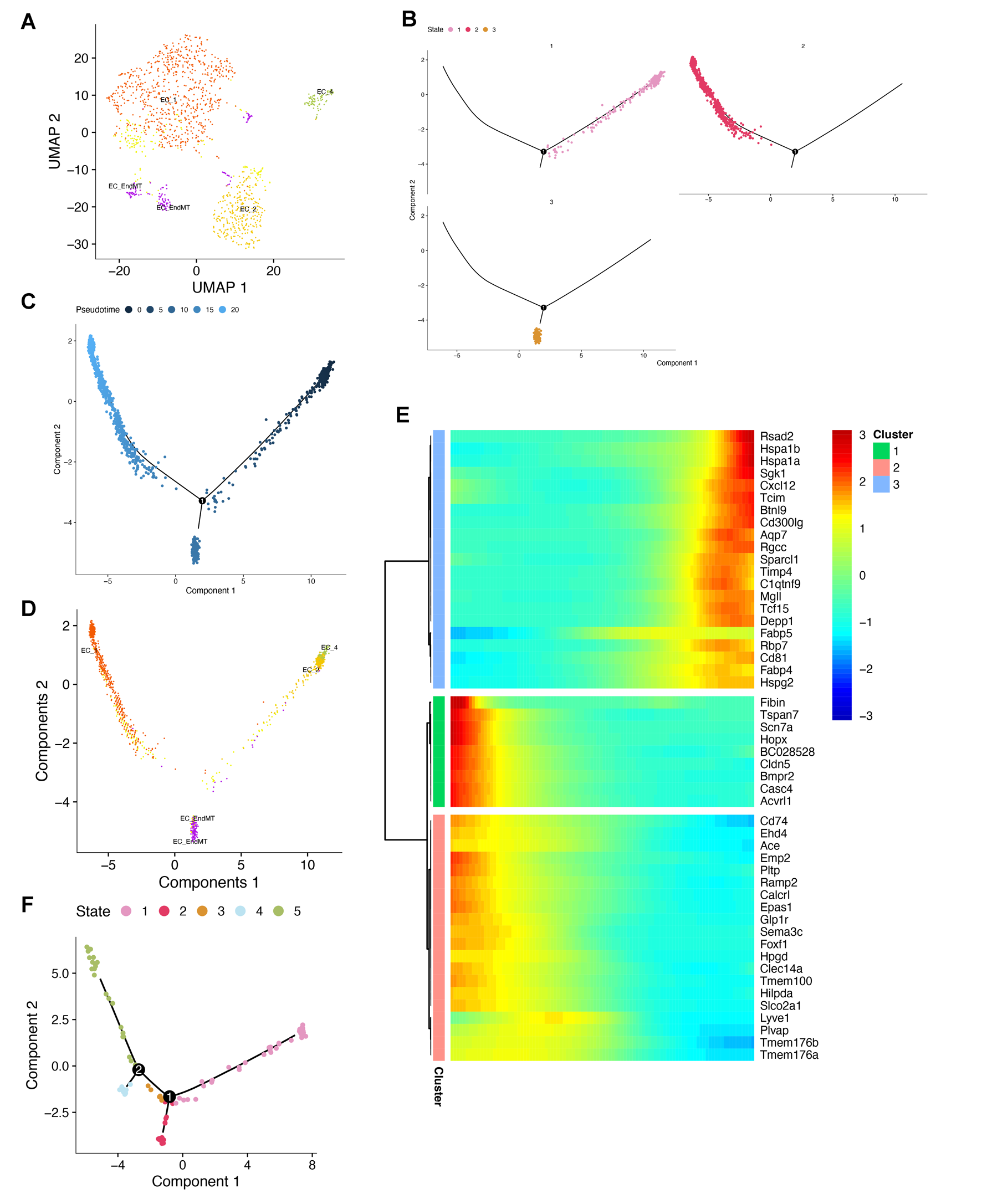

Supplement: Supplementary Figure 5 — Single-cell trajectories inferential analysis of endothelial cells. (A) UMAP plot showed individual cell types in cell clustering based on Monocle 2. (B) DDRtree plots showed the 3 cell states in endothelial cells. (C) DDRTree plot showed pseudotime values for endothelial cells. (D) The cell subtypes of endothelial cells in DDRtree plot. (E) Changes in expressions of the top 50 genes that were most associated with sorting of pseudotime. (F) DDRtree plot showed 5 cell states in the EC_EndMT. [file Image_5.TIF]
